# Association Between Serum Zinc Level and Comorbid Orthostatic Intolerance in Pediatric Patients with Migraine

**DOI:** 10.3390/nu17233753

**Published:** 2025-11-28

**Authors:** Sachi Tokunaga, Hideki Shimomura, Naoko Taniguchi, Yasuhiro Takeshima

**Affiliations:** Department of Pediatrics, Hyogo Medical University School of Medicine, Nishinomiya 663-8501, Japan; simo-ped@hyo-med.ac.jp (H.S.);

**Keywords:** migraine, zinc deficiency, orthostatic intolerance

## Abstract

**Background/Objectives:** Migraine affects approximately 10% of school-aged children, and frequently co-occurs with orthostatic intolerance (OI). However, although low serum zinc levels have been linked to migraine in adults, yet evidence in pediatric populations remains scarce. We aimed to investigate the association of serum zinc levels with comorbid OI in pediatric patients with migraine. **Methods:** We retrospectively analyzed medical records of 57 patients (26 male, 31 female; median age 13 years) newly diagnosed with migraine between December 2017 and March 2022. Diagnosis was made according to the International Classification of Headache Disorders, third edition criteria. Serum zinc, iron, copper, and ferritin concentrations were measured at initial presentation. Zinc deficiency was defined as serum concentration < 80 μg/dL. OI diagnosis required ≥2 characteristic symptoms based on modified Japanese diagnostic criteria. **Results:** The median serum zinc concentration was 80.7 μg/dL, with 40% of patients exhibiting zinc deficiency. Patients without comorbid OI (54%) demonstrated significantly lower serum zinc levels than those with OI (77.5 vs. 84.7 μg/dL, *p* < 0.001). Linear mixed model analysis identified comorbid OI as the only clinical factor significantly associated with serum zinc concentrations (*p* = 0.019). **Conclusions:** Pediatric patients with migraine without comorbid OI exhibit lower serum zinc levels than those with OI, suggesting differences in the underlying pathophysiology. Further large-scale, prospective studies including healthy control groups are warranted to validate these results.

## 1. Introduction

Migraine, a disabling neurological disorder, is a common health issue in children and adolescents, with a prevalence of approximately 10% among school-aged children [1]. Pediatric patients with migraine often experience reduced quality of life, affecting their physical health, education, and socialization [2].

An imbalance in serum trace elements, including zinc, copper and iron is involved in the pathophysiology of migraine [3,4,5]. Among these, iron has attracted particular attention owing to its role in neurotransmitter synthesis and inflammatory regulation, both of which may contribute to migraine mechanisms [6]. Several studies have reported a possible association between iron deficiency and migraine, particularly in women of reproductive age, who are at higher risk of iron deficiency due to menstrual blood loss [7]. Despite these findings, data on the relationship between iron status and migraine in pediatric populations remain limited.

Adult patients with migraine often exhibit low zinc levels [4,5]. Zinc, an essential trace element, has demonstrated anti-inflammatory effects [8]. Previous studies have examined the efficacy of zinc supplementation for migraine. Mazaheri et al. [9] and Ahmadi et al. [10] reported that zinc supplementation reduced the frequency, severity, and duration of migraine attacks. However, the subjects did not necessarily have zinc deficiency in both studies. In addition, while these studies have reported an association between migraine and zinc in adults, the children’s literature contains scarce reports on this topic.

Establishing a reference value for zinc is necessary to understand the association between zinc and migraine in children. However, no internationally recognized standard has been established for defining fasting serum zinc deficiency in children. Thus, there is a need for population-specific reference ranges to accurately assess zinc deficiency and its potential role in childhood migraine.

Children with migraine often have various comorbid conditions, including orthostatic intolerance (OI), neurodevelopmental disorders, epilepsy, and sleep disturbances [11]. OI, a common pediatric disorder, is characterized by difficulty tolerating an upright posture due to symptoms or signs that improve upon returning to a supine position [12]. OI includes conditions, such as orthostatic hypotension (OH), vasovagal syncope, and postural tachycardia syndrome (POTS) [12]. Therefore, accurately recognizing and diagnosing the presence of comorbid OI remains important [13]. We have previously reported that cyproheptadine was less effective in treating migraine associated with comorbid OI. This finding suggests that migraine with comorbid OI may have a different underlying pathophysiology compared to migraine without OI [14].

In this study, we first analyzed serum trace elements, including zinc, copper and iron as well as clinical factors in pediatric patients with migraine. Second, we investigated whether these factors were associated with comorbid OI.

## 2. Methods

### 2.1. Study Design and Participants

This retrospective study analyzed the medical records of patients newly diagnosed with migraine between December 2017 and March 2022. Diagnosis was made according to the International Classification of Headache Disorders, third edition (ICHD-3) [15]. Both migraine with and without aura were included. Data on patient age, sex, BMI, serum concentrations of zinc, iron, copper, and ferritin, as well as the presence of OI, were analyzed.

### 2.2. Evaluation

Serum concentrations of zinc, iron, copper, and ferritin were measured at the first visit. The timing of blood tests was not consistent. Blood samples were collected between migraine attacks in all patients, and none were taking zinc supplements. The Japanese Society of Clinical Nutrition guideline, defined serum zinc concentration < 80 μg/dL as zinc deficiency [16]. Based on that definition, we divided patients into two groups according their serum zinc concentrations: those with concentrations < 80 μg/dL, defined as zinc deficiency, and those with concentrations ≥ 80 μg/dL, defined as the normal range.

We defined OI as meeting ≥ 2 of the following major symptoms: susceptibility to vertigo and dizziness upon standing; fainting tendency in the standing position, which in severe cases leads to fall; nausea on taking a hot bath or encountering unpleasant experiences; palpitation and/or dyspnea after mild exercise; and difficulty getting out of bed. The diagnostic criterion was judged positive if the aforementioned symptoms were observed at least once per week. These criteria were based on the screening symptoms in the Japanese diagnostic criteria of orthostatic dysregulation (OD), with some modifications [17]. The original criteria define OD as fulfilling three or more major symptoms, two majors plus one or more minor symptoms, or one major plus three or more minor symptoms. The minor symptoms were as follows: pallor; anorexia; episodic umbilical colic, fatigability, frequent headache, and motion sickness [17]. Subtype classification requires an active standing test. In clinical practice, conditions are classified into four subtypes—OH, POTS, neutrally mediated syncope, and delayed orthostatic intolerance—based on the test results. For data analysis, we categorized patients based on the presence or absence of comorbid OI and zinc deficiency.

### 2.3. Statistical Analysis

Categorical variables were compared using Fisher’s exact test and numerical variables with Student’s *t*-test or Mann–Whitney U test. Spearman’s rank correlation coefficient (ρ) was used to assess the relationship between serum zinc and serum iron, copper, and ferritin. The variables age, iron level, and copper level were analyzed using Student’s *t*-test as a parametric method, while the non-normally distributed variables BMI, ferritin level, and zinc level were analyzed using Mann–Whitney U test as a non-parametric test. Linear mixed model (LMM) analysis was performed for all variables to determine factors affecting zinc level, including age; BMI; sex; serum levels of iron, copper, and ferritin; and the presence of comorbid OI. Age, BMI, and sex, as well as their interactions with iron, copper, and ferritin homeostasis, were included as covariates in the analysis because they are known to influence serum Zn level and could contribute to variability in pediatric populations [18,19,20]. Age was calculated in days for all statistical analyses. Prior to fitting the regression model, multicollinearity was assessed using the variance inflation factor (VIF). All VIF values were found to be below 5.0, indicating that multicollinearity was not a significant issue in the model. Normality of variables was tested using the Shapiro–Wilk method. We adopted an LMM to examine factors associated with the outcome. This decision addresses the violation of the independence assumption (Durbin–Watson statistic d = 0.42) caused by our clustered data structure. The subject ID was included as a random intercept to explicitly model the within-subject correlation, thereby satisfying the assumption of independent residuals and ensuring robust standard error estimation. Statistical analyses were performed using JMP Pro 18 software for Windows (SAS Institute Inc., Cary, NC, USA). *p*-value < 0.05 was considered statistically significant.

### 2.4. Ethical Considerations

This study was conducted in accordance with the Declaration of Helsinki and other relevant national regulations and guidelines. The requirement for informed consent was waived owing to the retrospective nature of the study. Information regarding this research was made publicly available on our institutional website to ensure the opportunity to decline participation. The study design was approved by the Review Board of Hyogo Medical University, Hyogo, Japan (approval number: 4135).

## 3. Results

The baseline and demographic characteristics of the patients are shown in Table 1. In total, 57 patients (26 male, 31 female) with a median age of 13 years (range: 7–19) were included. The median BMI was 17.1 kg/m^2^ (range: 14.1–28.0). Median serum concentrations of zinc, iron, copper and ferritin were 80.7 μg/dL (range: 57.8–113.3), 97 μg/dL (range: 51–158), 88 μg/dL (range: 52–133), and 53.4 ng/mL (range: 8.2–248), respectively. Zinc deficiency (serum zinc concentration < 80 μg/dL) was observed in 23 patients (40%), and normal zinc concentration (≥80 μg/dL) in 34 (60%). None of the patients had low iron concentration (<50 μg/dL), three had low copper concentrations (<70 μg/dL), and one a low serum ferritin concentration (<12 ng/mL). In total, 31 patients (54.4%) had comorbid OI. Serum trace elements and ferritin concentrations were presented separately in males and females; however, no significant differences were observed between the sexes (Table 2).

Analysis of the relationship between each trace element and serum zinc concentration revealed that no significant correlation was observed between serum zinc and serum iron (ρ = −0.09, *p* = 0.95), copper (ρ = −0.2, *p* = 0.14), or ferritin (ρ = 0.16, *p* = 0.23). We evaluated the demographic characteristics of the patients between normal range and deficiency of their serum zinc level. Patients with zinc deficiency had significantly lower age (*p* < 0.001) and BMI (*p* = 0.048) and were significantly more likely not to have comorbid OI (*p* < 0.001) (Table 3).

We also evaluated the demographic characteristics based on the presence or absence of comorbid OI (Table 4). The migraine-without-comorbid OI group had significantly lower age and serum zinc levels and a significantly higher prevalence of zinc deficiency. The LMM analysis showed that comorbid OI was the only factor statistically associated with zinc level (*p* = 0.019; Table 5).

## 4. Discussion

In this study, we analyzed serum zinc concentrations in pediatric patients with migraine. In the analysis of all patients, the median serum zinc concentration was within the normal range (80.7 μg/dL). Further stratified analysis revealed that patients with migraine without comorbid OI showed significantly lower zinc levels, whereas those with comorbid OI maintained normal zinc levels. These findings may provide valuable insights into the differing pathophysiology based on OI comorbidity status and the role of zinc in pediatric migraine.

Many studies have suggested a possible link between iron deficiency and migraine, particularly in adult women of reproductive age [7]. Compared with controls, female patients exhibited significantly higher rates of iron deficiency anemia and lower serum ferritin levels, whereas no significant differences were observed among males [7]. Fallah et al. demonstrated that in pediatric patients, iron deficiency was associated with increased monthly migraine frequency, severity, and related disability. They also reported that iron therapy significantly reduced headache frequency, severity, duration, and related disability scores in this population [21]. No patients in this study had iron deficiency, and only one showed reduced ferritin levels. While patient age, sex, and study region may have influenced the findings, further research is needed to investigate the relationship between iron deficiency and migraines in pediatric populations.

There are few reports on the effect of serum copper levels on migraines. Some studies have reported that serum copper levels are lower during interictal periods [5] compared with controls, whereas others observed no differences during attacks [4]. However, other studies have shown a positive correlation between migraine severity and serum copper levels [22]. In summary, existing research on the relationship between copper and migraine remains conflicting. In the present study, a decline in serum copper levels was observed only in three cases, highlighting the need for further investigation.

Zinc deficiency is strongly associated with stunting, respiratory infections, diarrhea, and dermatitis [23]. However, headaches are generally not listed as symptoms of zinc deficiency. Zinc is essential for the growth and development of young children [23]. Therefore, most current studies on zinc deficiency in children focus on those under the age of 5 years, with limited research on older children. Although previous research has reported reference intervals of serum zinc concentrations in children, the findings vary widely depending on the study population and methodology. Reported median or mean serum zinc concentrations include 62.7 μg/dL (mean, Thailand) [24], 72.7 μg/dL (median, Turkey) [25], 88.39 μg/dL (median, China) [20], 89 μg/dL (mean, America) [26], and 107.23 μg/dL (mean, Iran) [27]. Developing countries, particularly in South and Southeast Asia, sub-Saharan Africa, and Central America, have been identified as regions at high risk for inadequate zinc intake [28]. Age, BMI, and dietary habits have also been reported to influence serum zinc levels [20]. Given this considerable variability, reference intervals for serum zinc concentrations in children have not been clearly established and may require age- and country-specific definitions. Therefore, we used adult reference values in the present study, acknowledging that pediatric-specific ranges remain to be determined.

Low serum zinc levels have been reported in adult patients with migraine [4,5]. Gonulla et al. revealed significantly lower serum zinc levels in patients with migraine compared to healthy controls during the ictal period [4], while Silva et al. observed similar findings during the interictal period [5]. In contrast, evidence on serum zinc levels in pediatric patients with migraine remains limited. In the current study, the initial analysis of the entire patient cohort regardless the comorbid OI showed the median serum zinc level within the normal range. However, additional stratified analysis revealed that serum zinc levels were low in patients with migraine without comorbid OI. Therefore, although migraine comorbid with OI meets the ICHD-3 diagnostic criteria for migraine, it has been suggested that the underlying mechanism of headache development in these patients may differ from that driving migraine without OI.

Comorbid conditions are a major challenge in the treatment of pediatric migraine. Reported comorbid conditions include OI, neurodevelopmental disorders, epilepsy, and sleep disturbances [11]. OI is one of the most common comorbid conditions in pediatric patients with migraine. Patients with OI typically manifest vertigo and dizziness when standing, fainting in the standing position, nausea, and palpitations [17]. They also develop headaches. However, their detailed manifestations are not reported or classified as secondary headaches in ICHD-3. This may be attributed to the lack of standardized global diagnostic criteria for OI. Some classifications include OH and POTS under OI [12,29], while others categorize them under orthostatic dysregulation (OD) [17]. In this study, the diagnosis of OI was based solely on symptoms, and the use of the active standing test was left to the discretion of each attending physician. Therefore, not all patients underwent the test. When pharmacological treatment was considered, the active standing test was often performed because it influences therapeutic decision-making. As a result, detailed subclassification into OH or POTS could not be consistently applied. This limitation prevented a detailed diagnosis based on blood pressure and pulse rate. However, these conditions are believed to share the underlying pathology, characterized by reduced effective circulatory blood volume or imbalanced blood flow distribution. Therefore, we adopted a symptom-based diagnostic approach following the Japanese criteria without setting specific thresholds for blood pressure or heart rate. The lack of standardized diagnostic criteria for OI, OH, and POTS remains a significant barrier to research. Establishing international diagnostic standards and incorporating new objective indicators are essential for ensuring more consistent diagnoses.

In the present study, median serum zinc levels were normal in all patients with migraine; however, in the group without comorbid OI, the median serum zinc concentration was low at 77.5 μg/dL. Previous studies have reported that female patients with arterial hypotension and propensity for orthostatic fainting exhibited higher lymphocyte zinc levels than controls which may contribute to a tendency toward OH [30]. Because OH is classified as a type of OI, we believe that pediatric patients with migraine and comorbid OI have relatively higher serum zinc levels than those without OI. Thus, serum zinc levels may differ between migraine cases with and those without comorbid OI, suggesting possible differences in their underlying pathophysiology. Migraine and OI can present individually or as comorbidities in pediatric patients, and their symptoms often overlap. However, this comorbid presence is frequently overlooked [13]. Recognizing and appropriately diagnosing each condition is crucial, because their treatments differ [13]. In general, migraine and OI are diagnosed through a detailed history and physical examination, including an orthostatic standing test [13]. Additionally, considering serum zinc levels may be useful for differentiating between the two conditions. Specifically, lower serum zinc levels may indicate a higher likelihood of migraine without comorbid OI. As the participants in this study were limited to patients who met the diagnostic criteria for migraine, we were unable to examine the clinical significance of OI beyond its coexistence. Future analyses are needed to determine whether these findings can be applied to predict headache frequency, severity, or treatment response.

This study has some limitations. First, the research does not include a control group of healthy children for comparison, necessary to confirm that serum zinc levels are indeed low in patients with migraine without comorbid OI. Second, the diagnosis of OI as a comorbid condition was based solely on symptom-based clinical assessment, making it difficult to elucidate the underlying pathophysiology. In addition, the definition and diagnostic criteria for OI differ across countries, limiting the comparability or generalizability of our findings. Future investigations should incorporate standardized orthostatic testing, such as active standing test and tilt-table testing, to allow for more accurate pathological assessment. Third, serum zinc levels exhibit diurnal fluctuations, with values tending to be higher in the morning and lower in the afternoon [31]. They also tend to decrease after meals [32]. Therefore, measurement is recommended early in the morning on an empty stomach. However, owing to the retrospective study design, the timing of blood sampling was inconsistent. Regarding statistical analysis, while robust LMMs were employed to manage data dependencies, we recognize that our analytical approach involved multiple hypothesis tests. We chose not to apply formal adjustments for multiple comparisons, acknowledging that this decision carries an inherent risk of type I error inflation (false positives). Consequently, all findings should be subject to cautious interpretation, and their definitive validation is contingent upon replication in subsequent independent research.

Future studies should use standardized, fasting morning samples to minimize variability in serum level. Furthermore, several important variables that could influence serum zinc level were not assessed in this study. These include pubertal status and age-related hormonal changes, dietary habits and zinc intake, use of supplements and medication, inflammatory status, migraine frequency and chronicity, recent migraine attacks, and socioeconomic or environmental factors. Incorporating questionnaire surveys into future prospective studies to assess these potential confounding factors could provide more comprehensive data on the determinants of serum zinc levels and help clarify their relationship with migraine and comorbid OI. Moreover, a controlled trial of zinc supplementation may be a feasible next step. The optimal dosage and duration of supplementation should be guided by established pediatric nutritional recommendations.

## 5. Conclusions

Low serum zinc levels may differ between pediatric migraine cases with and without comorbid OI, suggesting possible differences in the underlying pathophysiological mechanisms and may potentially informing targeted treatment approaches. While our results provide preliminary evidence for zinc’s potential role in pediatric migraine, particularly in those without OI, larger prospective studies with standardized measurement protocols and control groups are needed to validate these observations and explore their clinical implications.

## Figures and Tables

**Table 1:** Baseline and demographic characteristics of the participants (*n* = 57).

Characteristic	Value
Median age, years (range)	13 y 0 m (7 y 3 m–19 y 9 m)
Sex, *n* (%)	
Male	26 (45.6)
Female	31 (54.4)
BMI (kg/m^2^), median (range)	17.1 (14.1–28.0)
Zn (μg/dL), median (range)	80.7 (57.8–113.3)
Distributions of serum zinc concentration, *n* (%)	
<60 μg/dL	1 (2)
60 ≤ x < 80 μg/dL	22 (38)
≥80 μg/dL	34 (60)
Fe (μg/dL), median (range)	97 (51–158)
Cu (μg/dL), median (range)	88 (52–133)
Ferritin (ng/mL), median (range)	53.4 (8.2–248)
Classifications	
Migraine without OI, *n* (%)	31 (54.4)
Migraine with OI, *n* (%)	26 (45.6)

Abbreviations: BMI, body mass index; Cu, copper; Fe, iron; m, month; OI, orthostatic intolerance; y, year; Zn, zinc.

**Table 2:** Trace elements and ferritin by sexes.

	Male	Female	*p*-Value
Zn (μg/dL), median (range)	80.9 (67.2–113.3)	80.7 (57.8–106.2)	0.775
Fe (μg/dL), median (range)	100 (53–147)	95 (51–158)	0.531
Cu (μg/dL), median (range)	84.5 (52–133)	90.0 (67–132)	0.538
Ferritin (ng/mL), median (range)	52.9 (15.8–248)	54.3 (8.2–110)	0.791

Abbreviations: Zn, Zinc; Fe, Iron; Cu, Copper. Mann–Whitney U test.

**Table 3:** Characteristics of the participants (*n* = 57) based on serum zinc concentration.

Characteristic		Zn Deficiency (Zn < 80 µg/dL) (*n* = 23)	Normal Range (Zn ≥ 80 µg/dL) (*n* = 34)	*p*-Value
Sex	Male, *n*	8	18	0.278 **
	Female, *n*	15	16	
Median age, years (range)		10 y 4 m (7 y 6 m–14 y 8 m)	13 y 2 m (9 y 3 m–19 y 9 m)	<0.001 *
BMI (kg/m^2^), median (range)		16.6 (14.1–20.9)	17.8 (14.1–28.0)	0.048 *
Fe (μg/dL), median (range)		98 (53–158)	93.5 (51–158)	0.938 *
Cu (μg/dL) median (range)		89 (52–133)	85 (66–126)	0.114 *
Ferritin (ng/mL) median (range)		49 (16.2–110)	59.6 (8.2–248)	0.111 *
Comorbid OI, *n* (%)		2 (8.7)	24 (70.6)	<0.001 **

Abbreviations: BMI, body mass index; Cu, copper; Fe, iron; m, month; OI, orthostatic intolerance; y, year; Zn, zinc. * Mann–Whitney U test. ** Fisher’s exact test.

**Table 4:** Characteristics of the participants (*n* = 57) based on the presence or absence of comorbid OI.

Characteristic		Migraine Without OI (*n* = 31)	Migraine With OI (*n* = 26)	*p*-Value
Sex	Male, *n*	11	15	0.115 **
	Female, *n*	20	11	
Median age, years, (range)		11 y 2 m (7 y 3 m–19 y 9 m)	13 y 4 m (9 y 3 m–16 y 2 m)	<0.001 *
BMI (kg/m^2^), median (range)		16.9 (14.1–21.7)	17.9 (14.1–28.0)	0.162 *
Zn (μg/dL), median (range)		77.5 (57.8–102.9)	86 (74.4–113.3)	<0.001 *
Zn deficiency, *n* (%)		21 (67.7)	2 (7.7)	<0.001 **
Fe (μg/dL), median (range)		95 (53–158)	100.5 (51–158)	0.771 *
Cu (μg/dL), median (range)		94 (52–133)	84.5 (66–126)	0.122 *
Ferritin (ng/mL), median (range)		53.4 (16.2–248)	57 (8.2–200)	0.211 *

Abbreviations: BMI, body mass index; Cu, copper; Fe, iron; m, month; OI, orthostatic intolerance; y, year; Zn, zinc. * Mann–Whitney U test. ** Fisher’s exact test.

**Table 5:** Linear mixed model for factors affecting serum zinc concentrations in the participants.

Factor	*B*	Std. Error	*t*	95% CI	*p*-Value
Age	0.00	0.00	0.7	(0.00–0.01)	0.487
Sex	−0.56	2.99	−0.19	(−6.56 to 5.44)	0.852
BMI	−0.29	0.60	−0.49	(−1.50 to 0.91)	0.626
Fe	−0.02	0.05	−0.43	(−0.13 to 0.08)	0.670
Cu	−0.05	0.12	−0.41	(−0.28 to 0.19)	0.681
Ferritin	0.01	0.04	0.19	(−0.07 to 0.09)	0.853
OI	−7.87	3.26	−2.42	(−14.42 to −1.32)	0.020

Abbreviations: BMI, body mass index; Cu, copper; Fe, iron; OI, orthostatic intolerance.

## Data Availability

The original contributions presented in this study are included in the article. Further inquiries can be directed to the corresponding author.

## Abbreviations

The following abbreviations are used in this manuscript:

BMI	Body mass index
GABA	γ-aminobutyric acid
ICHD-3	International classification of headache disorders, third edition
NMDA	*N*-methyl-D-aspartate
OD	Orthostatic dysregulation
OH	Orthostatic hypotension
OI	Orthostatic intolerance
POTS	Postural tachycardia syndrome
